# Increasing levels of circulating Th17 cells and interleukin-17 in rheumatoid arthritis patients with an inadequate response to anti-TNF-α therapy

**DOI:** 10.1186/ar3431

**Published:** 2011-07-30

**Authors:** Der-Yuan Chen, Yi-Ming Chen, Hsin-Hua Chen, Chia-Wei Hsieh, Chi-Chen Lin, Joung-Liang Lan

**Affiliations:** 1 Division of Allergy, Immunology and Rheumatology, Taichung Veterans General Hospital, No. 160, Section 3, Taichung-Kang Road, Taichung, 407, Taiwan; 2 Faculty of Medicine, National Yang-Ming University, No. 155, Sec. 2, Li-Nong Street, Taipei 112, Taiwan; 3 School of Medicine, Chung-Shan Medical University, No.110, Sec.1, Jianguo N.Rd., Taichung, 402, Taiwan; 4 Institute of Biomedical Science, National Chung-Hsing University, No.250, Kuo-Kuang Rd., Taichung, 402, Taiwan

## Abstract

**Introduction:**

The objective of this study was to investigate the effects of tumor necrosis factor (TNF)-α inhibitors on circulating T helper-type 17 (Th17) cells and Th17-related cytokines in patients with rheumatoid arthritis (RA).

**Methods:**

The frequencies of circulating Th17 cells and serum levels of Th17-related cytokines were determined using flow cytometry analysis and ELISA, respectively, in 48 RA patients both before (baseline) and six months after anti-TNF-α therapy. Therapeutic response was evaluated using European League Against Rheumatism (EULAR) response criteria.

**Results:**

Significantly higher baseline frequencies of circulating Th17 cells and serum levels of interleukin (IL)-6, IL-17, IL-21, IL-23 and TNF-α were observed in active RA patients than in 12 healthy controls (all *P *< 0.001). After anti-TNF-α therapy, 36 patients (75%) were EULAR responders (20 good responders and 16 moderate responders) and 12 (25.0%) were non-responders. The mean levels of circulating Th17 cells and IL-17 significantly decreased (1.13% vs. 0.79%; 43.1 pg/ml vs. 27.8 pg/ml; respectively, both *P *< 0.001) in parallel with clinical remission in responders. Levels of IL-6, IL-21, IL-23 and TNF-α were significantly decreased after anti-TNF-α therapy in responders. In contrast, the mean levels of circulating Th17 cells and IL-17 significantly increased after anti-TNF-α therapy (2.94% vs. 4.23%; 92.1 pg/ml vs. 148.6 pg/ml; respectively, both *P *< 0.05) in non-responders. Logistic regression analysis identified a high baseline level of IL-17 as a significant predictor of poor therapeutic response.

**Conclusions:**

The beneficial effect of anti-TNF-α therapy might involve a decrease in Th17-related cytokines in responders, whereas rising levels of circulating Th17-cells and IL-17 were observed in patients with an inadequate response to anti-TNF-α therapy.

## Introduction

Rheumatoid arthritis (RA) is characterized by the infiltration of macrophages and T cells into the joints, synovial hyperplasia, cartilage degradation and bone erosions [1]. Tumor necrosis factor (TNF)-α is a crucial inflammatory mediator in rheumatoid synovitis and subsequent tissue damage in RA [2,3]. Although TNF-α inhibitors can be an effective and well-tolerated therapy for RA patients [4-6], a significant proportion of patients do not acquire advantageous effects [7]. In addition, the effect of TNF-α inhibitors on the immune response has not been fully explored.

T helper-type 17 (Th17) cells, a novel and distinct subset of Th cell, can secrete interleukin (IL)-17 in humans [8-10]. Interleukin-17 is a pleiotropic cytokine that participates in tissue inflammation and destruction by inducing the expression of pro-inflammatory cytokines and matrix metalloproteases [8,11,12]. The frequencies of Th17 cells were found to increase in peripheral blood mononuclear cells (PBMCs) of RA patients compared to healthy controls [13,14]. An enhanced expression of IL-17 has been observed in the rheumatoid synovium [15] and synovial fluids of patients with early RA [16]. Interleukin-17 *in vitro *stimulates the production of TNF-α and IL-1β, and also synergizes with TNF-α to induce cartilage loss and promote osteoclastogenesis [17,18]. A recent study showed that Th17 cells, but not Th1 cells, cooperate with synovial fibroblasts in a pro-inflammatory feedback loop that drives chronic destruction in RA [19]. Moreover, IL-17 has become a new therapeutic target for animal models with collagen-induced arthritis (CIA) and human RA [20-22]. These observations suggest that Th17 cells and IL-17 critically contribute to synovitis and bone destruction associated with RA.

Recently, TNF-α was shown *in vitro *to drive the production of IL-17 with the ability to differentiate T cells towards a Th17 phenotype [23]. In a psoriasis-like skin inflammation model, TNF-α enhanced the expression of Th17-related cytokine genes during priming but suppressed these cytokine transcripts when present during re-stimulation [24]. In CIA, TNF-α inhibitors reduced the number of Th17 cells in pathologic joints despite an increase of Th17 cells in inguinal lymph nodes [25]. Taken together, these findings show that TNF-α blockade has paradoxical effects on the expression of Th17-related cytokines in animal models of autoimmune diseases.

In humans, an engineered p75 TNFRII dimer, etanercept, suppressed the gene expression levels of Th17-related cytokines including IL-6 and IL-23 in cutaneous lesions of psoriasis [26]. Kageyama *et al. *also reported a significant decrease in serum levels of IL-23 at three and six months after etanercept therapy in RA patients [27]. TNF-α inhibitor, adalmumab, reduced the frequency of circulating Th17 cells and serum IL-6 levels in RA patients [28]. However, a recent study showed that an increased frequency of circulating Th17 cells after TNF-α blockade is accompanied by a decrease in Th17-specific chemokine receptor expression in RA [29]. When taken together, these results reveal conflicting effects of TNF-α inhibitors on Th17 cells and IL-17 in humans.

In the present study, we attempted to determine whether or not the clinical response to anti-TNF-α therapy of RA patients led to changes in the levels of circulating Th17 cells and Th17-related cytokines, and we also investigated their clinical implication.

## Materials and methods

### Patients

A total of 48 consecutive patients (39 females and 9 males; mean age ± SD 50.1 ± 13.5 years), who fulfilled the 1987 revised criteria of the American College of Rheumatology for RA [30], were evaluated before and six months after anti-TNF-α therapy. All patients remained in active disease in spite of treatment with methotrexate (MTX) and other disease-modifying anti-rheumatic drugs (DMARDs), for whom anti-TNF-α therapy was initiated based on the British Society for Rheumatology guidelines [31]. Fourteen patients received etanercept at a dose of 25 mg twice weekly and 34 patients received adalimumab at a dose of 40 mg every other week in combination with a stable dose of MTX of 7.5 to 15 mg weekly. Corticosteroids (≦10 mg/day) and non-steroid anti-inflammatory drugs (NSAIDs) were allowed but were given at stable doses for at least four weeks before and during the six-month anti-TNF-α therapy. Disease activity was assessed by the 28-joint disease activity score (DAS28) [32]. The therapeutic response was evaluated six months after anti-TNF-α therapy was started, according to the European League Against Rheumatism (EULAR) response criteria [33]. The patients were categorized into good, moderate or non-responders based on the amount of change in the DAS28 and the level of DAS28 reached. Good responders are defined as patients who have a decrease in DAS28 from baseline (ΔDAS28) of >1.2 and a DAS28 at the sixth month of < 3.2; moderate responders have either ΔDAS28 of >1.2 and a DAS28 at the sixth month of ≧3.2 or ΔDAS28 of 0.6 to 1.2 and a DAS28 at the sixth month of < 5.1; and non-responders are those who have either ΔDAS28 of < 0.6 or a DAS28 at the sixth month of ≧5.1 [33]. To obtain a better analysis, we combined good responders and moderate responders into EULAR responders. Twelve age- and sex-matched healthy volunteers (10 females and 2 males, 47.6 ± 8.1 years), who had no rheumatic disease, were used as normal controls. Blood samples were collected at baseline (before starting anti-TNF-α therapy) and six months after anti-TNF-α therapy. The Ethics Committee of Taichung Veterans General Hospital approved this study and the written consent of each participant was obtained.

### Quantitation of circulating Th17cells using flow cytometry analysis

In order to detect circulating Th17 cells, phycoerythrin (PE)-conjugated anti-IL-17 (eBioscience, San Diego, CA, USA) and Phycoerythrin-Cyanin 5 (PC5)-conjugated anti-CD4 (Beckman Coulter, Marseilles, France) were quantified using flow cytometry according to the manufacturer's protocol and a technique previously described [34,35]. Briefly, aliquots of 1,000 μl of the sterile heparinized whole blood were stimulated with a combination of 25 ng/ml of phorbol myristate acetate and 1 μg/ml of ionomycin (Sigma, Deisenhofen, Germany) and cultured for one hour at 37°C in a humidified 5% CO_2 _incubator. Whole blood was treated with 10 μg/ml of Brefeldin A (Sigma, Germany) to inhibit intracellular protein transport. Activated cultures of blood samples were washed in wash buffer (phosphate buffered saline, 5% foetal bovine serum, 0.1% sodium azide; Merck, Darmstadt, Germany) and then stained with 20 μl of PC5-conjugated CD4-specific monoclonal antibody (mAb) (Beckman Coulter, Marseilles, France) for 15 minutes at room temperature (RT). Erythrocytes were lysed by adding 2 ml of fluorescence-activated cell sorter (FACS) lysing solution (Becton Dickinson, Lincoln Park, NJ, USA). After five minutes of incubation, the samples were centrifuged and washed with 0.1% BSA-PBS, and subsequently fixed with 100 μl Reagent 1 (Beckman Coulter, Marseilles, France) for 10 minutes. After washing, the pellet was incubated with 100 μl Reagent 2, saponin (Beckman Coulter, Marseilles, France) for five minutes at RT in the dark. The samples were washed twice with 0.1% BSA-PBS and then incubated with PE-conjugated IL-17-specific mAb (eBiosciences, San Diego, CA, USA) for 30 minutes at RT in the dark. An isotype control IgG1-PE (eBiosciences, USA) was used for the IL-17 staining at RT in the dark. After staining, the cells were washed and immediately analysed using flow cytometry (Beckman Coulter, USA). Lymphocytes were gated on the basis of forward- and side- scatter properties and at least 10,000 CD4^+ ^cells were analysed. The results were analysed using Expo32 software (Beckman Coulter, Miami, FL, USA).

### Determination of serum levels of Th17-related cytokines by ELISA

Serum levels of IL-6, IL-17, IL-21, IL-23 and TNF-α were determined in 48 RA patients at baseline and after six months of anti-TNF-α therapy, and in 12 healthy controls using enzyme-linked immunosorbent assay (ELISA) according to the manufacturer's instructions (eBiosciences, USA).

### Determination of serum levels of anti-cyclic citrullinated peptide (anti-CCP) antibody and rheumatoid factor (RF)-IgM

Determination of the anti-CCP antibody was performed by ELISA using a commercial kit (INOVA Diagnostics Inc., San Diego, CA, USA). A result was considered positive for anti-CCP antibodies if the titer was above 20 IU/ml. Serum levels of RF-IgM were measured by nephelometry (Dade Behring Inc., Newark, DE, USA). A result was considered positive for RF when the concentration was above 15 IU/ml.

### Statistical analysis

The results are presented as the mean ± SD or median (interquartile range, IQR). The non-parametric Mann-Whitney U test was used for between-group comparisons of serum levels of IL-6, IL-17, IL-21, IL-23 and TNF-α, and for the percentages of circulating Th17 cells. The correlation coefficient was obtained by the non-parametric Spearman's rank correlation test. The Wilcoxon signed rank test was used to compare the percentages of circulating Th17 cells and serum levels of Th17-related cytokines during follow-up for the RA patients after anti-TNF-α therapy. A probability of less than 0.05 was considered significant.

## Results

### Baseline characteristics of RA patients with a different therapeutic response

As illustrated in Table 1, the majority of RA patients were female and all patients had active disease (DAS28, mean ± SD, 7.22 ± 0.79) before starting anti-TNF-α therapy. After anti-TNF-α therapy, 36 (75.0%) patients were EULAR responders, including 20 (41.7%) good responders and 16 (33.3%) moderate responders, whereas 12 (25.0%) were non-responders. Although non-responders had higher erythrocyte sedimentation rates (ESR) and RF titers compared to responders, the differences did not reach statistical significance. There were also no significant differences in the baseline demographic data, positive rate of RF and anti-CCP antibodies, daily dose of corticosteroids, or the proportion of patients previously using DMARDs between responders and non-responders.

**Table 1:** Baseline clinical characteristics and laboratory findings in RA patients with different response to anti-TNF-α therapy

	**RA with anti-TNF-α Rx**.		Healthy control
	Moderate-good responders (*n *= 36)	Non- responders (*n *= 12)	(*n *= 12)
Mean age, years	50.4 ± 14.5	49.3 ± 10.8	47.6 ± 8.1
Female (%)	29 (80.6%)	10 (83.3%)	10 (83.3%)
Disease duration, years	5.44 ± 2.42	5.37 ± 2.37	NA
RF positivity (%)	30 (83.3%)	11 (91.6%)	NA
Anti-CCP positivity (%)	29 (80.6%)	10 (83.3%)	NA
ESR (mm/1^st ^hr)	48.9 ± 25.4	63.8 ± 24.4	NA
DAS-28	7.18 ± 0.74	7.33 ± 0.94	NA
Daily steroid dose (mg)	6.5 ± 2.1	6.9 ± 2.4	NA
TNF-α inhibitors			
Etanercept (*n *= 14)	10 (71.4%)	4 (28.6%)	NA
Adalimumab (*n *= 34)	26 (76.5%)	8 (23.5%)	NA
Used DMARDs			
Methotrexate	33 (91.7%)	11 (91.6%)	NA
Sulfasalazine	31 (86.1%)	10 (83.3%)	NA
Hydroxychloroqine	32 (88.9%)	10 (83.3%)	NA
Ciclosporine	14 (38.9%)	5 (41.7%)	NA

Data are presented as mean ± SD or number (percentage).

RA, rheumatoid arthritis; Anti-CCP, anti-cyclic citrullinated peptide antibodies; DAS28, disease activity score for 28-joints; DMARDs, disease-modifying anti-rheumatic drugs; ESR, erythrocyte sedimentation rate; NA, not applicable; RF, rheumatoid factor; TNF-α, tumor necrosis factor-α.

### The frequencies of circulating Th17 cells in RA patients before anti-TNF-α therapy

**Figure 1:**
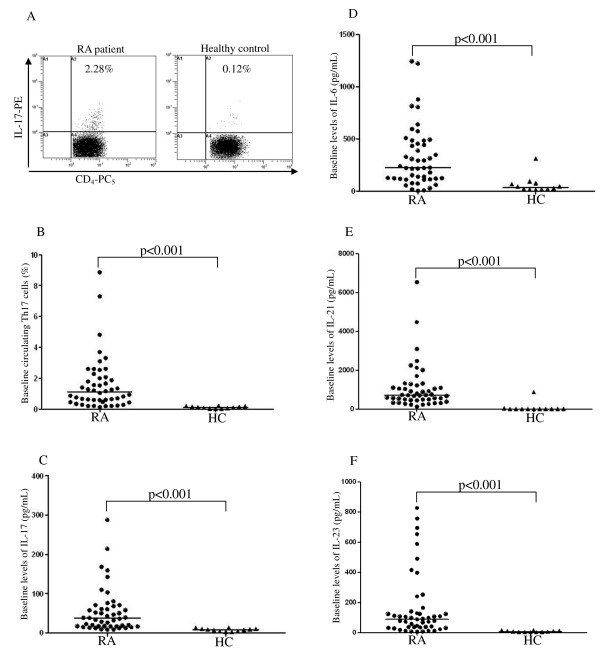
**The comparison of circulating levels of Th17-cells and Th17-related cytokines between RA patients and HC**. (**A**) Flow cytometric dot-plots of intracellular IL-17 production in Th cells obtained from peripheral blood of one representative patient with rheumatoid arthritis and healthy control. The comparison of circulating Th17 cells frequencies is shown (**B**) between 48 RA patients before starting anti-TNF-α therapy (baseline) and 12 HC. The comparison of baseline levels of serum IL-17 (**C**), IL-6 (**D**), IL-21 (**E**), and IL-23 (**F**) is shown between RA patients and HC. The horizontal line indicates median value. *P*-value was assessed by Mann-Whitney U test. HC, healthy control; IL, interleukin; RA, rheumatoid arthritis; Th17 cells, T helper-type 17 cells; TNF-α, tumor necrosis factor alpha.

**Figure 2:**
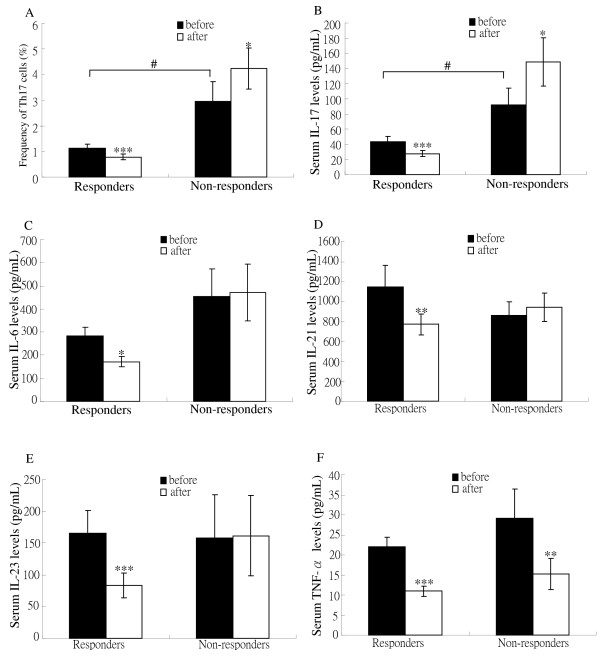
**The change in circulating levels of Th17-cells and Th17-related cytokines in EULAR responders and non-responders**. The changes in (**A**) the frequencies of circulating Th17-cells (T helper-type 17 cells) and serum levels of (**B**) interleukin (IL)-17, (**C**) IL-6, (**D**) IL-21, (**E**) IL-23, and (**F**) tumor necrosis factor (TNF)-α in 36 responders and 12 non-responders to anti-TNF-α therapy according to European League Against Rheumatism (EULAR) response criteria. Bars represent the mean value and SEM. *P < 0.05, **P < 0.005, ***P < 0.001, versus before starting anti-TNF-α therapy, determined by the Wilcoxon signed rank test.#*P *< 0.01, non-responders versus responders.

Representative examples of flow cytometric dot-plots of intracellular IL-17 staining in Th cells obtained from the PB of one RA patient and from a healthy control are shown in Figure 1 A. Significantly higher baseline frequencies of circulating Th17 cells were observed in RA patients (median 1.11%, IQR 0.50% to 2.05%) than in healthy controls (median 0.12%, IQR 0.05% to 0.18%; *P *< 0.001, Figure 1B). Among the RA patients, significantly higher baseline frequencies of circulating Th17 cells were observed in EULAR non-responders than in responders (*P *< 0.01, Figure 2A).

### Serum levels of Th17-related cytokines in RA patients before anti-TNF-α therapy

As shown in Figure 1, the baseline serum levels of Th17-related cytokines including IL-17, IL-6, IL-21 and IL-23, were significantly higher in RA patients than in healthy controls. Among the RA patients, significantly higher IL-17 levels were observed in non-responders than in responders (*P *< 0.01, Figure 2 and Table 2). Although non-responders seemed to have higher levels of IL-6 and TNF-α when compared to the levels in responders, the differences did not reach statistical significance. There was also no significant difference in the levels of Th17-related cytokines between the etanercept-treated group and the adalimumab-treated group (data not shown).

**Table 2:** Changes in levels of Th17-cells and Th17-related cytokines in RA patients with different therapeutic responses

	Moderate-good responders (*n *= 36)	Non-responders (*n *= 12)
Th17 cells (%)		
Before Rx.	1.13 ± 0.16	2.94 ± 0.77#
After Rx.	0.79 ± 0.11***	4.23 ± 0.80*
IL-17 level (pg/ml)		
Before Rx	43.1 ± 7.0	92.1 ± 22.2#
After Rx	27.8 ± 3.8***	148.6 ± 31.5*
IL-6 level (pg/ml)		
Before Rx	282.9 ± 37.1	453.7 ± 120.9
After Rx	171.7 ± 22.2*	471.6 ± 124.0
IL-21 level (pg/ml)		
Before Rx	1147.0 ± 216.3	863.5 ± 133.9
After Rx	769.8 ± 104.7**	942.9 ± 140.4
IL-23 level (pg/ml)		
Before Rx	166.1 ± 35.1	158.4 ± 67.6
After Rx	83.3 ± 19.7***	161.3 ± 63.3
TNF-α level (pg/ml)		
Before Rx	22.0 ± 2.4	29.2 ± 7.2
After Rx	11.0 ± 1.3***	15.2 ± 3.9**
RF titer (IU/ml)		
Before Rx	116.6 ± 32.7	368.7 ± 257.9
After Rx	101.2 ± 28.2	440.0 ± 324.4
Anti-CCP titer (IU/ml)		
Before Rx	58.7 ± 9.6	72.4 ± 22.0
After Rx	51.8 ± 8.7**	68.8 ± 20.3

Data are presented as mean ± SEM; RA, rheumatoid arthritis. *P < 0.05, **P < 0.005, ***P < 0.001, versus before anti-TNF-α treatment in each group

#*P *< 0.01, versus moderate-good responders.

Anti-CCP, anti-cyclic citrullinated peptide antibodies; IL, interleukin; RF, rheumatoid factor; Rx, treatment; Th17, type 17 helper T cell; TNF-α, tumor necrosis factor-α.

### Correlation between the frequencies of circulating Th17 cells and disease activity or serum levels of Th17-related cytokines in RA patients

The frequencies of circulating Th17 cells were positively correlated with DAS28 scores (r = 0.294, *P *< 0.05), IL-17 levels (r = 0.737, *P *< 0.001), IL-6 levels (r = 0.347, *P *< 0.05), and TNF-α levels (r = 0.495, *P *< 0.001) in RA patients before the anti-TNF-α therapy was started. Serum IL-6 levels were also positively correlated with IL-17 levels (r = 0.303, *P *< 0.05) in RA patients. After six months of anti-TNF-α therapy, the frequencies of circulating Th17 cells remained positively correlated with DAS28 scores (r = 0.375, *P *< 0.01), IL-17 levels (r = 0.727, *P *< 0.001), IL-6 levels (r = 0.311, *P *< 0.05), and TNF-α levels (r = 0.362, *P *< 0.05).

### Effects of TNF-α inhibitors on the levels of Th17 cells and Th17-related cytokines

As illustrated in Figure 2 and Table 2, the mean levels of circulating Th17 cells and IL-17 significantly declined after six-month anti-TNF-α therapy (1.13% vs. 0.79%; 43.1 pg/ml vs. 27.8 pg/ml; respectively, both *P *< 0.001), in parallel with the decrease in DAS28 (7.18 vs. 5.21, *P *< 0.001) and in anti-CCP titers (58.7 ± 9.6 vs. 51.8 ± 8.7 IU/ml, *P *< 0.005) in responders. Serum levels of IL-6, IL-21, IL-23 and TNF-α were also significantly decreased after anti-TNF-α therapy in responders. In contrast, the mean levels of circulating Th17 cells and IL-17 significantly increased (2.94% vs. 4.23%; 92.1 pg/ml vs. 148.6 pg/ml; respectively, both *P *< 0.05), while TNF-α levels decreased after anti-TNF-α therapy (29.2 pg/ml vs. 15.2 pg/ml, *P *< 0.005) in non-responders. There were no significant changes in RF titers after anti-TNF-α therapy in either responders or non-responders.

### Multiple logistic regression analysis of the effects of Th17 cell frequencies and serum Th17-related cytokines on therapeutic efficacy

Multiple logistic regression analysis showed that only a high baseline IL-17 level (≧40.0 pg/ml) could significantly predict a poor response to anti-TNF-α therapy (*P *< 0.01), with a medium level of specificity (83.3%) and sensitivity (66.7%).

## Discussion

Consistent with the findings of previous reports [36,37], our results showed that 36 (75.0%) patients have moderate or good EULAR response to the six-month anti-TNF-α therapy and 12 (25.0%) were non-responders. Although non-responders seem to have higher baseline ESR, titers of RF and anti-CCP antibodies, and serum levels of TNF-α and IL-6, when compared to responders, the differences do not reach statistical significance. Our findings suggest that an inadequate response to anti-TNF-α therapy is not merely a reflection of high disease activity.

The present study is the first attempt to investigate the effects of TNF-α inhibitors on the levels of circulating Th17 cells and Th17-related cytokines in RA patients with a different therapeutic response. In order to obtain a better reflection of *in vivo *cytokine patterns than is achievable with PBMCs, whole blood was stimulated with mitogens and lymphocytes double-stained with IL-17 and CD4 were analysed using flow cytometry. Our results showed significantly higher baseline frequencies of circulating Th17 cells in active RA patients compared with healthy controls, confirming the findings of previous studies [13,14,29]. We also showed that the baseline levels of circulating Th17 cells were positively correlated with DAS28 scores in RA patients. Our data and the findings of previous studies [15,16] suggest a potential role of Th17 cells in the pathogenesis of RA.

Similar to the results of recent studies [28], our findings showed a significant decrease in the frequencies of circulating Th17 cells after anti-TNF-α therapy, in parallel with the decrease in DAS28 in EULAR responders. Although the diseases studied were different, our results were also consistent with the findings of previous studies which showed that the TNF-α inhibitor reduced Th17 cell responses with the amelioration of psoriatic skin lesions [38] and that the TNFR1 inhibitor suppressed the Th17 response with clinical benefits in experimental autoimmune encephalomyelitis mice [39]. In contrast, our results showed a significant increase in circulating Th17 cell frequencies in non-responders. Our findings in non-responders were similar to the results of a recent study that showed an increased frequency of circulating Th17 cells after TNF-α blockade in RA patients [29]. The divergent effects on Th17 cells *in vivo *after anti-TNF-α therapy for RA patients suggest that there may be different subsets of the immune response to TNF-α inhibitors. It is tempting to speculate that the change in frequencies of circulating Th17 cells may help to differentiate between EULAR responders and non-responders because these changes occur within the first six months of anti-TNF-α therapy.

The Th17 cells have a specific role in immune function through the production of effector cytokines. Through the secretion of IL-17, Th17 cells act on the differentiation of osteoclasts and bone resorption [40], and can stimulate the monocytes to produce pro-inflammatory cytokines, thus amplifying the inflammatory cascade [11,17,18]. In the present study, we showed that serum IL-17 levels were significantly elevated and correlated with DAS28 in active RA patients. Our data were consistent with the results of recent studies describing an increase in IL-17 levels in RA patients [14,41], and they also supported the results of clinical trials showing the therapeutic benefits of IL-17 blockade [20-22]. During a longitudinal follow-up of RA patients who received anti-TNF-α therapy, we found that serum IL-17 levels significantly decreased, in parallel with the clinical remission in the responders, whereas increasing IL-17 levels were found in non-responders. Whether or not this imbalance of Th17 cells and IL-17 between PB and affected joints contributes to the change in Th17-related cytokines after anti-TNF-α therapy remains unclear.

Accumulating evidence indicates that IL-6 can enhance Th17 cell differentiation by promoting the sequential engagement of IL-21/IL-23 pathways and that it plays a critical role in Th17-dependent autoimmune diseases [42]. In the present study, we showed that the high baseline levels of IL-6 were positively correlated with the frequencies of circulating Th17 cells in RA patients. After anti-TNF-α therapy, a significant decrease in the levels of circulating Th17 cells and IL-17, in parallel with the decrease in DAS28 and serum IL-6 levels was observed in responders. These observations support the role of IL-6 in the differentiation of Th17 cells [42-44], and the therapeutic benefits of IL-6 receptor inhibitors in RA patients [45].

Interleukin-21 is required to reinforce differentiation of Th17 cells, and it plays a critical role in Th17-dependent autoimmune diseases [46]. In contrast to IL-12, IL-23 does not promote the development of Th1 cells, but it is crucial for the expansion and maintenance of Th17 cells [47]. In the present study, we showed that the baseline levels of IL-21 and IL-23 were significantly elevated in active RA patients, supporting their role in the pathogenesis of this disease [42,48]. In addition, we found that serum levels of IL-21 and IL-23 significantly decreased, in parallel with the clinical remission in responders after anti-TNF-α therapy. Consistent with our data, the results of the study by Kageyama *et al. *also showed a significant decrease in serum IL-23 levels at three and six months after anti-TNF-α (etanercept) therapy in RA patients [27].

The mechanisms of the therapeutic effects of TNF-α inhibitors on RA have not been fully explored. Lundy *et al. *showed that TNF-α has a positive feedback effect on Th17 cells, causing a vicious cycle of synovitis [49]. TNF-α also promotes IL-17 production by inducing dendritic cells to direct the differentiation of Th cells towards the Th17 phenotype [23], and by the administration of TNF-like ligand 1A enhancing Th17 differentiation with IL-17 production [50]. Our results showed a significant decrease in the levels of circulating Th17 cells and TNF-α after effective therapy with TNF-α inhibitors, suggesting that the down-regulation of both Th17-related cytokines and TNF-α may be one of the mechanisms of anti-TNF-α therapy [19]. Our findings support the hypothesis that anti-TNF-α therapy could reduce Th17 responses by blocking the TNF-α-dependent positive feedback system [23]. Another explanation may be the inhibition of emigration of Th17 cells from lymphoid organs after anti-TNF-α therapy [24].

In the present study, we showed that one-fourth of RA patients have an inadequate response to anti-TNF-α therapy despite a significant decrease in serum TNF-α level, suggesting that neutralization of TNF-α cannot fully explain the therapeutic effect of TNF-α inhibitors in non-responders. Additionally, significantly higher baseline levels of circulating Th17-cells and IL-17 were observed in non-responders when compared to responders. During a longitudinal follow-up of RA patients undergoing anti-TNF-α therapy, we found a significant increase in circulating levels of Th17-cells and IL-17 in non-responders. Although the cut-off value for baseline IL-17 (40 pg/mL) used in our study was not absolute, a multivariate logistic regression analysis showed that only a high baseline level of IL-17 could be a significant predictor of poor therapeutic response to TNF-α inhibitors. Our observations suggest that an inadequate response to anti-TNF-α therapy may reflect TNF-independent but Th17-dominant inflammatory process, which has been observed in previous studies [15,16].

This was a preliminary study that enrolled a limited number of active RA patients who were followed up for six months. Because we mainly investigate the effects of anti-TNF-α therapy on Th17 cells and Th17-related cytokines, we did not include a group of RA patients without anti-TNF-α therapy as a disease control. Because the patients enrolled in our study were not an early RA population, our results might not be directly applicable to early RA patients undergoing anti-TNF-α therapy. Therefore, a long-term study enrolling a larger group of patients, including an additional early RA population, or a control group using DMARDs only, is required to validate these findings. Although the erroneous increase in IL-17 levels may have been due to the heterophilic binding by serum RF [51,52], there were no significant differences in RF positivity or titers between responders and non-responders in the present study.

## Conclusions

Our results show that anti-TNF-α therapy not only neutralizes the effects of TNF-α, but it also down-regulates the IL-6/Th17 axis in EULAR responders. In contrast, anti-TNF-α therapy causes a significant increase in Th17 cells in RA patients with an inadequate response to anti-TNF-α therapy. A high baseline level of IL-17 may have a predictive value for poor therapeutic response to TNF-α inhibitors. Although the small sample size of this study did not allow for a definitive conclusion, our findings at least suggest that Th17-dependent inflammation is implicated in the pathogenesis of a subset of RA patients with poor response to TNF-α inhibitors, and that treatment targeting IL-17 may be beneficial for these patients [21,22].

## Abbreviations

Anti-CCP: anti-cyclic citrullinated peptide; CIA: collagen-induced arthritis; DAS28: 28-joint disease activity score; DMARDs: disease-modifying anti-rheumatic drugs; ELISA: enzyme-linked immunosorbent assay; ESR: erythrocyte sedimentation rate; EULAR: European League Against Rheumatism; IL: interleukin; MTX: methotrexate; NSAIDs: non-steroid anti-inflammatory drugs; PBMCs: peripheral blood mononuclear cells; PC5: Phycoerythrin-Cyanin 5; PE: phycoerythrin; RA: rheumatoid arthritis; RF: rheumatoid factor; RT: room temperature; Th17 cells: T helper-type 17 cells; TNF-α: tumor necrosis factor-α.

## Competing interests

The authors declare that they have no competing interests.

## Authors' contributions

DYC and JLL participated in the study design, acquisition of data, interpretation of results, and manuscript preparation. YMC participated in the study design, acquisition of data, interpretation of results, and assisted in drafting the manuscript. HHC, CWH and CCL contributed to analysis and interpretation of data, and assisted in drafting the manuscript. All authors approved the final manuscript.
